# Mucin (Muc) expression during pancreatic cancer progression in spontaneous mouse model: potential implications for diagnosis and therapy

**DOI:** 10.1186/1756-8722-5-68

**Published:** 2012-10-26

**Authors:** Satyanarayana Rachagani, María P Torres, Sushil Kumar, Dhanya Haridas, Michael Baine, Muzafar A Macha, Sukhwinder Kaur, Moorthy P Ponnusamy, Parama Dey, Parthasarathy Seshacharyulu, Sonny L Johansson, Maneesh Jain, Kay-Uwe Wagner, Surinder K Batra

**Affiliations:** 1 Department of Biochemistry and Molecular Biology, University of Nebraska Medical Center, Omaha, NE, 68198-5870, USA; 2 Eppley Institute for Research in Cancer and Allied Diseases, University of Nebraska Medical Center, Omaha, NE, 68198-5870, USA; 3 Department of Pathology and Microbiology, University of Nebraska Medical Center, Omaha, NE, 68198-5870, USA

**Keywords:** Mucins, Inflammatory cytokines, Kras^G12D^ mouse model

## Abstract

**Background:**

Pancreatic cancer (PC) is a lethal malignancy primarily driven by activated *Kras* mutations and characterized by the deregulation of several genes including mucins. Previous studies on mucins have identified their significant role in both benign and malignant human diseases including PC progression and metastasis. However, the initiation of MUC expression during PC remains unknown because of lack of early stage tumor tissues from PC patients.

**Methods:**

In the present study, we have evaluated stage specific expression patterns of mucins during mouse PC progression in (Kras^G12D^;Pdx1-Cre (KC)) murine PC model from pancreatic intraepithelial neoplasia (PanIN) to pancreatic ductal adenocarcinoma (PDAC) by immunohistochemistry and quantitative real-time PCR.

**Results:**

In agreement with previous studies on human PC, we observed a progressive increase in the expression of mucins particularly Muc1, Muc4 and Muc5AC in the pancreas of KC (as early as PanIN I) mice with advancement of PanIN lesions and PDAC both at mRNA and protein levels. Additionally, mucin expression correlated with the increased expression of inflammatory cytokines *IFN-γ* (p < 0.0062), *CXCL1* (p < 0.00014) and *CXCL2* (p < 0.08) in the pancreas of KC mice, which are known to induce mucin expression. Further, we also observed progressive increase in inflammation in pancreas of KC mice from 10 to 50 weeks of age as indicated by the increase in the macrophage infiltration. Overall, this study corroborates with previous human studies that indicated the aberrant overexpression of MUC1, MUC4 and MUC5AC mucins during the progression of PC.

**Conclusions:**

Our study reinforces the potential utility of the KC murine model for determining the functional role of mucins in PC pathogenesis by crossing KC mice with corresponding mucin knockout mice and evaluating mucin based diagnostic and therapeutic approaches for lethal PC.

## Introduction

Pancreatic cancer (PC) has an extremely poor prognosis with a five year survival rate of less than 6% [1] and a median survival of approximately 5-6 months after being diagnosed. This high mortality rate of PC is due to its late clinical presentation with approximately 80% of the patients having metastatic disease at the time of diagnosis [2]. Further, PC exhibits an unusual resistance to current chemo- and radiotherapies, which are mainly directed for palliative care [3]. Early detection of PC remains a clinical challenge because of its silent nature, retroperitoneal location, small size of precursor lesions and unavailability of early stage tissue and serum samples from PC patients.

Molecules that are specifically overexpressed in tumor tissues not only serve as useful diagnostic markers but also as potential targets for therapeutic intervention. Serum-based molecular markers such as cancer antigen 125 (CA125), antigen SC6 (SC6-Ag), pyruvate kinase isoenzyme type 2 (M2-PK), macrophage inhibitory cytokine 1 (MIC-1) [4] and the most commonly used PC marker CA19-9 lack sensitivity, specificity or reproducibility and hence cannot be used routinely for diagnosing PC. Due to unavailability of early stage PC tissues, genetically engineered mouse models of PC progression serve as a reliable source of early stage lesions and serum samples and can potentially help in understanding the molecular alterations at the earliest stages of the disease for identifying potential biomarkers and novel targets for therapeutic intervention.

Mucins are high molecular weight glycoproteins that form a physical barrier to protect the epithelial cells under normal physiological conditions. However, alterations in mucin expression, localization or glycosylation patterns have been associated with cancer development and contribute to enhanced transformation, cancer cell growth, and decreased immune surveillance [5,6]. Further, due to their aberrant overexpression in several epithelial malignancies [7,8], mucins are recognized as attractive targets for therapy and diagnosis [9]. Our previous studies have established that human PC is characterized by an altered pattern of mucin expression at different stages of tumor progression [10,11].

MUC1, MUC4, MUC5AC are the most differentially overexpressed mucins in human PC [8,10,11]. While MUC4 and MUC5AC are undetectable in benign pancreatic diseases and normal pancreas [10,11], their expression increases progressively with the advancement of PC to an extent that both genes are among the top differentially overexpressed genes in PC [12]. Importantly, overexpression of MUC1, MUC4 and MUC5AC are associated with poor survival [13,14] and serve as potential tumor markers for PC [15]. MUC1 is a transmembrane glycoprotein that is expressed in normal pancreas [16] but overexpressed and aberrantly glycosylated in >90% of metastatic PDAC and its aberrant expression has been associated with increased metastasis and poor prognosis of PC and other cancers [8,17-19]. Knock-down of MUC1 and MUC4 expression decreases growth and metastatic potential of PC cells indicating that mucins play a functional role in PC progression [5,20,21].

While mucins have been studied extensively in late stage clinical samples and PC cell lines, limited information is available on early stage lesions of PC because precursor lesions observed in patient samples are in tandem with the aggressive form of the disease. Thus, mucin expression in these early lesions is suggestive but not definitive as an early event in PC. Due to the lack of availability of early-stage tissues and samples from patients, the expression profiles of mucins and their true potential as early biomarkers of PC remains to be tested. Since MUC1, MUC4 and MUC5AC have considerable homology with their murine counterparts [22-25], the present study was aimed to determine the expression profile of Muc1, Muc4 and Muc5ac in Kras^G12D^ spontaneous mouse model for PC. This mouse model closely recapitulate the genetic and histopathological features of human PC, and therefore it can potentially help in understanding the molecular alterations at earliest stages of the malignant disease for identifying potential biomarkers and novel therapeutic targets. Hence, they serve as suitable preclinical models to evaluate therapeutic and preventive strategies and provide a rare opportunity to identify and validate mucin based early biomarkers for PC [26].

## Methods

### Experimental animals

The B6.129-Kras^tm4Tyj^ (01XJ6) and B6. FVB-Tg (Ipf1-cre)1Tuv (01XL5) mice were obtained from the NCI Mouse Models of Human Cancers Consortium (MMHCC) (Frederick, MD, USA). These animals (LSL-Kras^G12D^ and Pdx1-Cre) were crossed to remove the LSL cassette in order to activate *Kras*^*G12D*^ (Kras^G12D^;Pdx1-Cre/floxed Kras^G12D^) allele in the pancreas of the mouse. The F1 progeny was genotyped for *Kras* as well as *Pdx1-Cre* by using specific primers for *Kras* and *Pdx1-Cre* by Polymerase chain reaction (PCR). Animals that were positive for *Kras*^*G12D*^ and *Pdx1-Cre* expressed the mutated *Kras*^*G12D*^ allele in the pancreas. The floxed *Kras*^*G12D*^ animals (positive for both *Kras* and *Pdx1-Cre*) and their contemporary littermates positive for either *LSLKras*^*G12D*^ or *Pdx1-Cre* were euthanized at 7, 10, 25, 30, 40 and 50 weeks of age (eight animals/group/time point). Throughout the experiment, animals were provided with food and water *ad libitum* and subjected to a 12-h dark/light cycle. Animal studies were performed in accordance with the U.S. Public Health Service “Guidelines for the Care and Use of Laboratory Animals” under an approved protocol by the University of Nebraska Medical Center Institutional Animal Care and Use Committee (IACUC).

### DNA isolation and genotyping

Animals were tail clipped at 10-14 days of age and DNA was isolated using standard protocol (Maxwell 16 mouse tail DNA purification kit, Promega, Madison, WI, USA). The genotyping of *Kras* and *Pdx1-Cre* was performed by PCR using the following primer sequences *Kras* K006F-5’-CCT TTA CAA GCG CAC GCA GAC TGT AGA-3’, *Kras* K005R-5’- AGC TAG CCA CCA TGG CTT GAG TAA GTC TGC A-3’ and *Pdx1-Cre* F-5’-CTG GAC TAC ATC TTG AGT TGC -3’ and *Pdx1-Cre* R-5’-GGT GTA CGG TCA GTA AAT TTG -3’. The PCR amplification reaction contained 1 μl of genomic DNA (100 ng), 0.3 μl 10 pmol of each primer, 10 μl of 2X PCR master mix (DNA Polymerase, 400 μM each of dATP, dGTP, dCTP, dTTP and 3 mM MgCl_2_) and 8.4 μl of autoclaved water. PCR amplification was carried out in a programmable thermal cycler (MJ Research, Minnesota, USA) using the following program: denaturation for 5 min at 95°C, followed by 40 cycles of amplification by denaturation for 1 min at 94°C, annealing at 2 min at 59°C, elongation for 45 sec at 72°C and a final extension of 10 min at 72°C. The PCR products were resolved on 1.5% agarose gel to confirm the genotype of each animal based on the amplification of target regions.

### Isolation of RNA

Total RNA was isolated from the pancreas of floxed *Kras*^*G12D*^ (Kras^G12D^;Pdx1-Cre) and unfloxed *Kras*^*G12D*^ (LSLKras^G12D^) by using the mirVana™ miRNA Isolation Kit (Applied Biosystems/Ambion, Austin, TX, USA). RNA concentration was measured by using a NanoDrop^TM^ Spectrophotometer (NanoDrop Technologies Inc., Wilmington, DE, USA), and the quality was analyzed with a bioanalyzer (Agilent technologies, Waldbronn, Germany). Samples with good integrity were used for cDNA synthesis.

### cDNA synthesis and real time PCR

Total RNA was isolated from the pancreas and the cDNA was synthesized by reverse transcription. Reverse transcription of RNA was performed by adding 10 μl of (2000 ng) total RNA, 1 μl of Oligo (dT) and 1 μl of 10 mM dNTP incubated at 65°C for 5 min and immediately chilled on ice. Then, the master mix containing the following components were added (4 μl of (5X) first strand RT buffer, 1 μl of 0.1 M DTT, 1 μl of RNaseOUT (RNase Inhibitor) and incubated at 42°C for 2 min. Finally, 1 μl (50 units) of SuperScript II RT was then added to each tube mix, and incubated at 42°C for 50 min followed by 70°C for 15 min in order to destroy the superscript II RT (Invitrogen, Carlsbad, CA, USA).

Real time primers for all the mouse genes (*Muc1, Muc4, Muc5AC, IFN-γ, CXCL1,* and *CXCL2*) were designed using Primer 3 software (Table 1). Real-time PCR was performed on the Light cycler 480 II PCR System, (Roche Applied Science, Indianapolis, IN, USA). Real-time PCR reactions were performed in triplicate, and non-template controls (NTCs) and standard curve were run for each assay under similar conditions. Real time PCR was performed in a 10 μl reaction volume containing 5 μl 2X SBYR green Master mix (Roche applied science, Indianapolis, IN, USA), 3.2 μl of autoclaved nuclease free water, 1 μl of diluted RT product (1:10) and 0.2 μl each of forward and reverse primer (5pmol/μl). The cycling conditions were as follows: 95°C for 10 min, followed by 40 cycles of 95°C for 15 sec and 60°C for 1 min. Gene expression levels were normalized to the level of *β-actin* expression and were reported relative to the expression level in RNA from corresponding normal controls.

**Table 1:** Real time PCR primer sequences

**Gene**	**Primer Sequence**
*IFN-γ*	For: 5’-ACTGGCAAAAGGATGGTGAC-3’
	Rev: 5’-TGAGCTCATTGAATGCTTGG-3’
*CXCL-1*	For: 5’-CTTGCCTTGACCCTGAAGC-3’
	Rev: 5’-AGGTGCCATCAGAGCAGTCT-3’
*CXCL-2*	For: 5’-TCAAGAACATCCAGAGCTTGAG-3’
	Rev: 5’-TTCAGGGTCAAGGCAAACTT-3’
*Muc5AC*	For: 5’-CCTCTCAGAGGAATGTGACTCTGCGC-
	3’Rev:5’-CCAGGCAGCCACACTTCTCAACCT-3’
*mMuc4*	For: 5’-GAGGGCTACTGTCACAATGGAGGC-3’
	Rev:5’-AGGGTTCCGAAGAGGATCCCGTAG-3’
*mMuc1*	For: 5’-CCCTACCTACCACACTCACGGACG-3’
	Rev:5’-GTGGTCACCACAGCTGGGTTGGTA-3’

### Antibodies

Anti-mouse Muc1 (mouse monoclonal antibody recognizing the cytoplasmic tail of Muc1), and Anti-mouse Muc5AC (mouse monoclonal) antibody were purchased from Abcam® (Cambridge, MA, USA). The anti-Muc4 (4A-rabbit polyclonal) antibody used in this study was designed by us and developed by GenScript (Piscataway, NJ, USA). Rabbits were immunized with a 15 amino-acid peptide specific to the tandem repeat region of mouse Muc4 (CAGYRPPRPAWTFGD). Analysis of tissue sections pre-incubated with the blocking peptide was conducted in order to confirm the specificity of the antibody.

### Hematoxylin and eosin staining (H&E)

The F1 progeny of (N=8) floxed *Kras*^*G12D*^ (Kras^G12D^;Pdx1-Cre) and unfloxed *Kras*^*G12D*^ (LSLKras^G12D^) animals were sacrificed at 7, 10, 25, 30, 40 and 50 weeks of age. A section of the pancreas from these animals was fixed in 10% formalin (Fisher Scientific, Fair Lawn, NJ, USA). The tissues were then embedded in paraffin and serial tissue sections (4 μm thick) were cut. The sections were deparaffinized using EZ-DeWaxTM (Bio genex, San Roman CA, USA) and dehydrated gradually. Subsequently, the sections were stained with hematoxylin and eosin (H&E) stains and examined under a light microscope as described [27].

### Immunohistochemistry (IHC) analysis

Pancreatic tissues isolated from transgenic mice of different ages were embedded in paraffin after being fixed in 10% formalin for at least 48 h. Subsequently, 4 μm sections of paraffin-embedded pancreas were sliced and prepared for histological analysis. After placing the slides in an oven at 56°C overnight, these were deparaffinized after washing several times in xylene (Fisher Scientific, Fair Lawn, NJ, USA). Tissues were then rehydrated with decreasing concentrations of ethanol. After incubating the tissues for 30 min in the presence of 5% H_2_O_2_ in methanol to block the endogenous peroxidase, tissue sections were blocked in 2.5% horse serum for 2 h. Without washing the tissue sections, the corresponding primary antibodies were added at the optimum concentrations, which were determined after standardization experiments. The corresponding dilutions used in these sections were: 1:200 anti-Muc1, 1:4000 anti-Muc4, 1:400 anti-Muc5AC. Following overnight incubation, sections were washed three times with PBST and the horseradish peroxidase-conjugated secondary antibody was added for 30 min. IHC staining of the respective mucins were developed after colorimetric detection with a 3,3’-diaminobenzidine (DAB) reagent kit (Vector Laboratories, Burlingame, CA, USA) followed by hematoxylin staining. Tissues were then dehydrated with increasing concentrations of ethanol followed by a xylene wash. IHC staining was evaluated by a pathologist after mounting with Permount mounting medium (Fisher Scientific, Fair Lawn, NJ, USA). Expression of each mucin was scored on a scale of 0–3 where 0-negative, 1-weak, 2-moderate and 3- represented strong immunoreactivity to the antibody used. Further the percentage of cells positive for the antibody was scored on a scale of 1–4 where 1: 0–25% cells positive; 2: 26–50% positive; 3: 51–75% positive; and 4: 76–100% positive. The composite score was then obtained by multiplying the staining intensity and the percentage of immunoreactive cells and it ranged from 0 to 12.

### Statistical analyses

Fold change in the mRNA expression of various genes were calculated by ΔΔCt method. Mouse *β-actin* was used for normalization. A change of 2 fold or more (on the log scale 0.3 or more) was considered statistically significant. A Student’s *t*-test was used to calculate the significance in the staining pattern for each mucin at different stages of PC progression. All *p-*values <0.05 were considered statistically significant.

## Results

### Pancreatic cancer progression

**Figure 1:**
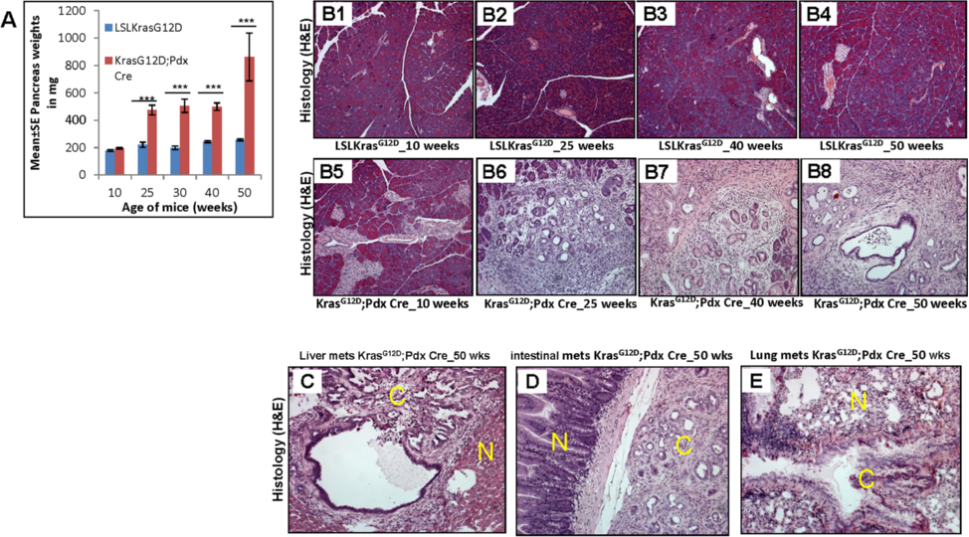
**Changes in the pancreas weight and histology during the progression of pancreatic cancer in Kras**^**G12D**^**mouse model.** (**A**) Weight of pancreas during the mice pancreatic cancer progression in Kras^G12D^ transgenic mouse model compared to LSL-Kras^G12D^ mice (*** p-value <0.0001). (**B1**-**B4**) Light microscopic pictures (100x) of H&E stained pancreatic sections from unfloxed Kras^G12D^ (LSL-Kras^G12D^) at 10, 25, 40 and 50 weeks of age, respectively. (**B5**-**B8**) Light microscopic pictures (100x) of H&E stained pancreatic sections showing PanIN lesions from floxed Kras^G12D^ (Kras^G12D^;Pdx1-Cre) at 10, 25, 40 and 50 weeks of age, respectively. (**C**, **D**, **E**) Metastatic lesions from liver, small intestines and lung stained with H&E (100x).

The floxed *Kras*^*G12D*^ animals (i.e. positive for both *Kras*^*G12D*^ and *Pdx1-Cre*) and their contemporary littermates harboring either *LSLKras*^*G12D*^ or *Pdx1-Cre* were euthanized at 7, 10, 25, 30, 40 and 50 weeks of age (N = 8 for each time point) and individual pancreas was resected and weighed. The average weight of the pancreas in the Kras^G12D^;Pdx1-Cre animals was significantly higher (p < 0.0001) than those of age-matched LSLKras^G12D^ control animals. Importantly, the average pancreas weight increased from 25 weeks (475 mg) to 50 weeks (863 mg) of age in Kras^G12D^;Pdx1-Cre while no significant change was observed in control animals (Figure 1A). These differences in the pancreas weight suggested the occurrence of pathological changes in Kras^G12D^;Pdx1-Cre mice.

Upon microscopic examination of the H&E stained tissue sections, no lesions were observed in the pancreas of LSLKras^G12D^ mice (Figure 1B1-1B4), while Kras^G12D^;Pdx1-Cre mice pancreas showed the presence of PanIN lesions as early as 10 weeks of age, which progressively developed into PDAC by 50 weeks of age (Figure 1B5-1B8). Specifically, at 10 weeks of age, mostly PanIN-I lesions were observed (Figure 1B5), which progressed to PanIN-II and III lesions at 25 weeks of age, replacing the majority of pancreatic parenchyma (Figure 1B6). At 40 weeks of age, the majority of parenchyma was replaced by advanced PanIN III lesions and extensive desmoplasia (Figure 1B7), and at 50 weeks of age, the pancreas parenchyma was replaced with PDAC (Figure 1B8). Metastatic lesions involving liver, lung and small intestines were observed at 50 weeks of age in 60-70% of the Kras^G12D^;Pdx1-Cre mice (Figure 1C-1E).

### Muc1 expression during pancreatic cancer progression in Kras^G12D^ mouse model

**Figure 2:**
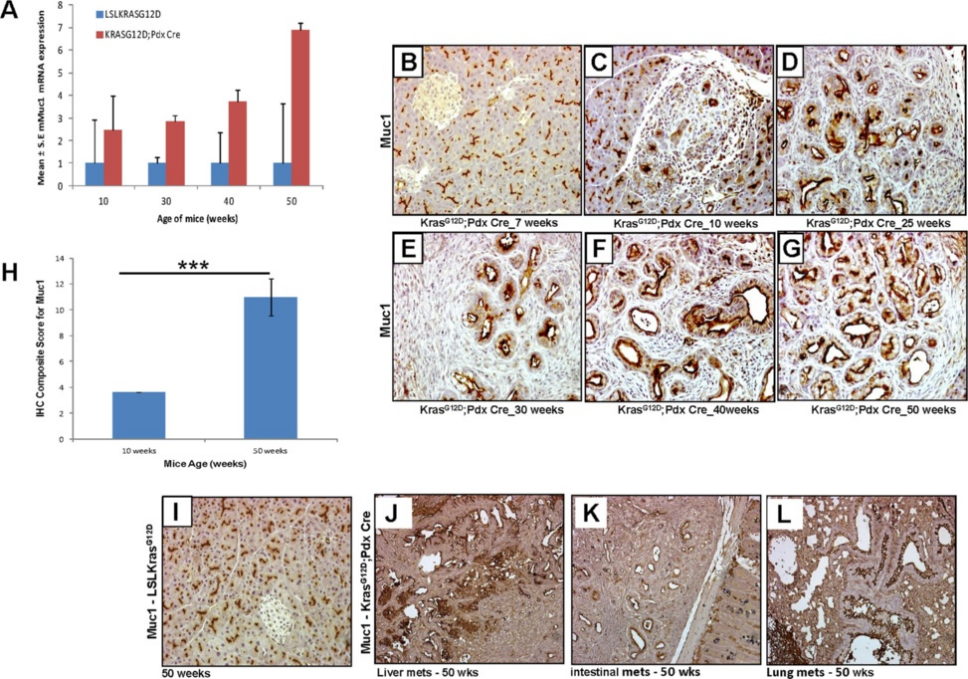
**Expression pattern of Muc1 during the progression of pancreatic cancer in Kras**^**G12D**^**mouse model.** (**A**) *Muc1* mRNA expression was determined by quantitative real time PCR. (**B**, **C**, **D**, **E**, **F**, and **G**) Muc1 protein expression during the progression of pancreatic cancer in Kras^G12D^ mouse model was analyzed by IHC. The formalin fixed pancreatic tissue collected during mouse PC progression (10 weeks to 50 weeks) were paraffin embedded and 4 μm tissue sections were cut and stained with anti-Muc1 antibody. Light microscopic pictures (200x) are shown. (**H**) Composite scores of pancreatic tissues of KrasG^12D^;Pdx1-Cre mice stained with anti-Muc1 antibody. (**I**) Normal pancreas isolated from 50 week old mice showing expression of Muc1 in normal ducts. (**J**, **K**, **L**) Muc1, expression in the metastatic lesions involving liver, small intestines and lungs, respectively isolated from 50 week Kras^G12D^ mice.

Previous reports have shown that MUC1 is overexpressed during the progression of human PC and it plays an important role in cancer invasion and metastasis [6]. In this study, real time-PCR analysis showed an increase in the expression of *Muc1* from 10 weeks (fold change 2.5) to 50 weeks (fold change 6.9) of age in the pancreas of Kras^G12D^;Pdx1-Cre mice in comparison to the LSLKras^G12D^ control mice (Figure 2A). The pancreas of unfloxed *Kras*^*G12D*^ mice expressed basal level of *Muc1* (Figure 2A). IHC analysis showed an elevated protein expression of Muc1 in the pancreas of Kras^G12D^;Pdx1-Cre mice starting from 10 weeks of age (Figure 2B-2G). The intensity of Muc1 expression increased in pancreatic tissues isolated from 10 weeks to 50 weeks of age with an increase in composite score from 3.6 to 11 (p < 0.0001) (Figure 2H). Muc1 protein was predominately localized at the membrane of pancreatic ductal cells. The IHC results are in agreement with real time-PCR data, as a basal level expression of Muc1 was observed in the pancreas of unfloxed LSLKras^G12D^ mice, which did not increase even in 50 weeks old mice (Figure 2I). Further, Muc1 expression was also observed in the metastatic lesions involving liver, small intestines and lungs at 50 weeks of age in Kras^G12D^;Pdx1-Cre animals (Figure 2J-2L).

### Expression of Muc4 during pancreatic cancer progression in Kras^G12D^ mouse model

**Figure 3:**
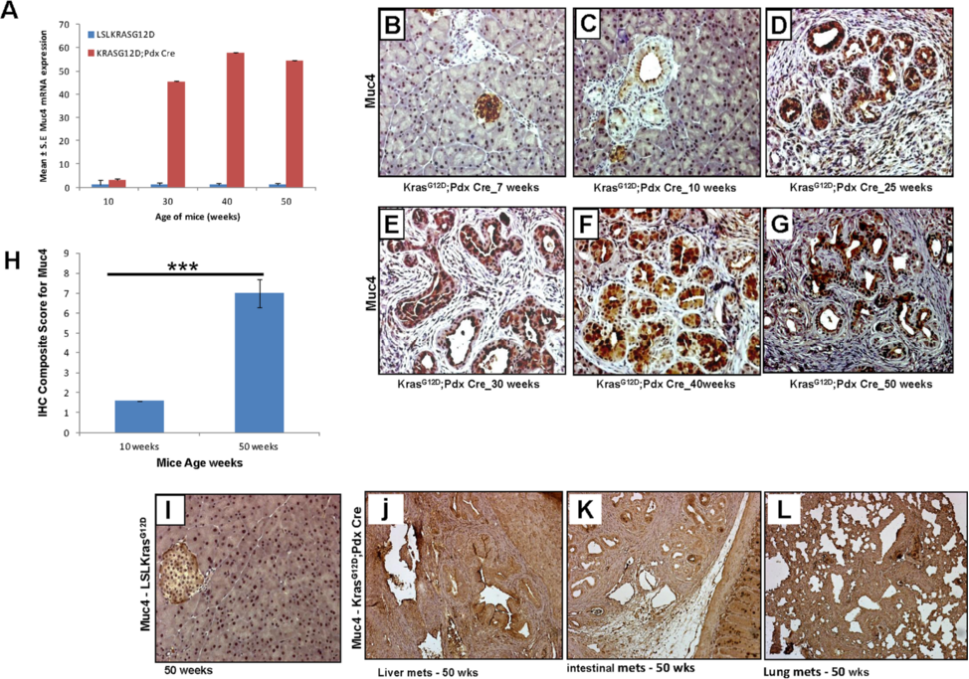
**Expression pattern of Muc4 during the progression of pancreatic cancer in Kras**^**G12D**^**mouse model.** (**A**) *Muc4* mRNA expression was determined by quantitative real time PCR. (**B**, **C**, **D**, **E**, **F**, and **G**) Muc4 protein expression during the progression of pancreatic cancer in Kras^G12D^ mouse model (**H**) Composite scores of pancreatic tissues of Kras^G12D^;Pdx1-Cre mice stained with Muc4 antibody. (**I**) Normal pancreas from 50 week old mice were negative for Muc4 expression. (**J**, **K**, **L**) Muc4 expression in the metastatic lesions involving liver, small intestines and lungs, respectively in 50 weeks old Kras^G12D^ mice.

Previous studies from our lab have shown that MUC4 is aberrantly overexpressed in human PC [6] and has a role in the progression and metastasis of PC cells [5,20,28]. We determined the expression pattern of Muc4 glycoprotein during the initiation and progression of PC in the Kras^G12D^;Pdx1-Cre mouse model (10-50 weeks) by real time-PCR and IHC. A significant increase in *Muc4* transcripts was observed in the pancreas of Kras^G12D^;Pdx1-Cre mice from 10 (fold change 2.9) to 50 weeks (fold change 54) of age (Figure 3A). Similar to normal human pancreas, no expression of *Muc4* was observed in the pancreas of LSLKras^G12D^ mice. Similarly, IHC analysis showed a progressive increase in Muc4 protein levels in the pancreas of Kras^G12D^;Pdx1-Cre mice from 7 to 50 weeks of age (Figure 3B-3G). These results were in agreement with real time-PCR results as there was a significant (p < 0.0001) increase in the composite score for Muc4 expression in the pancreas of Kras^G12D^;Pdx1-Cre mice from 1.6 at 10 weeks to 7.0 by 50 weeks of age (Figure 3H). Muc4 expression was observed in both membrane and cytoplasm of pancreatic ductal cells associated with PanIN lesions, while no expression was detected in the adjoining acinar and stromal cells. The pancreas of LSLKras^G12D^ mice was completely negative for Muc4 even at 50 weeks of age (Figure 3I). High expression of Muc4 was also observed in the metastatic lesions involving small intestines as well as liver and lungs of 50 weeks old Kras^G12D^;Pdx1-Cre mice (Figure 3J-3L).

### Expression of Muc5ac during pancreatic cancer progression in Kras^G12D^ mouse model

**Figure 4:**
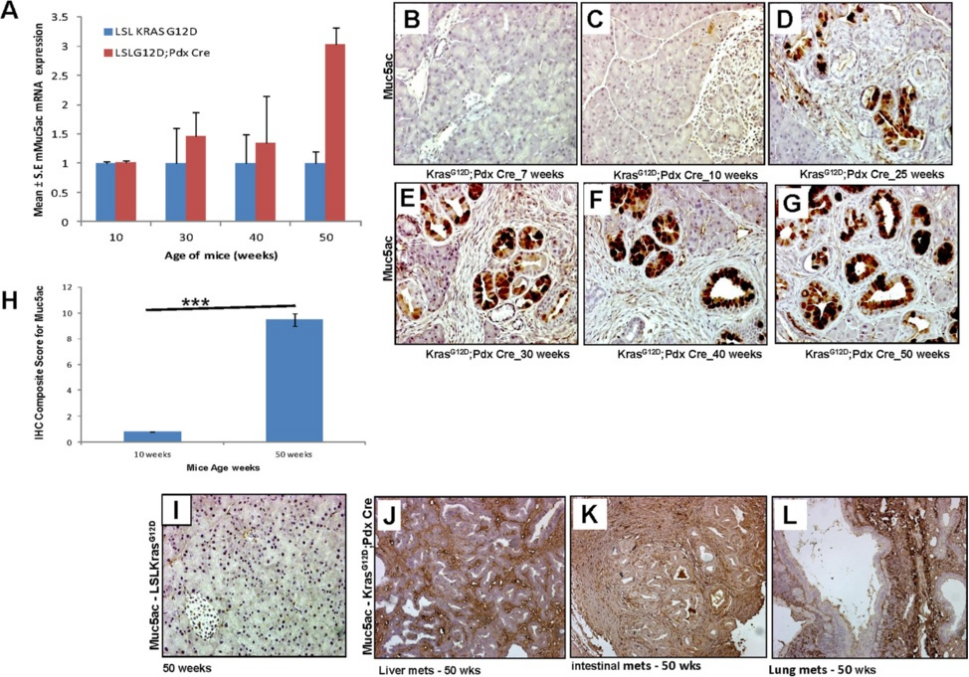
**Expression pattern of Muc5AC during the progression of pancreatic cancer in Kras**^**G12D**^**mouse model.** (**A**) Muc5AC mRNA expression was determined by quantitative Real Time PCR. (***p-value =0.0002) (**B**, **C**, **D**, **E**, **F**, and **G**) Muc5AC protein expression during the progression of pancreatic cancer in Kras^G12D^ mouse model was analyzed by IHC. (**H**) Composite scores of pancreatic tissues of Kras^G12D^;Pdx1-Cre mice stained with Muc5AC antibody. (**I**) Normal pancreas from control mice (50 weeks) was stained negative for Muc5ac expression. (**J**, **K**, **L**) Muc5AC expression in the metastatic lesions involving liver, small intestines and lungs, respectively, in Kras^G12D^;Pdx1-Cre mice.

It has been previously established that the expression of MUC5AC, a gel-forming secretory mucin increases in tandem with the increase in grade of PanIN lesions and PDAC. However no expression of MUC5AC has been detected in the normal human pancreas [19,29]. In the present study, real time-PCR analysis showed an increase in the expression of *Muc5AC* in the pancreas of Kras^G12D^;Pdx1-Cre mice from 10 weeks (fold change 1.2) to 50 weeks (fold change 3.0) of age when compared to LSLKras^G12D^ mice (Figure 4A). Real time-PCR analysis in the pancreas of LSLKras^G12D^ mice showed no change in the expression of *Muc5AC* across the different age groups (Figure 4A). Similarly, IHC analysis showed a gradual increase in the protein expression of Muc5AC in the pancreas of Kras^G12D^;Pdx1-Cre mice (Figure 4B-4G). The composite scores for Muc5AC expression in pancreatic tissues increased from 0.8 (i.e. no expression) at 10 weeks of age to 9.5 (p < 0.0001) in 50 weeks old Kras^G12D^;Pdx1-Cre mice. No expression of Muc5AC was detected in the pancreas of age-matched unfloxed LSLKras^G12D^ mice (Figure 4I). The IHC analysis of metastatic lesions involving liver, small intestines and lungs at 50 weeks of age showed strong Muc5AC expression (Figure 4J-4L).

### Inflammation during the progression of pancreatic cancer

**Figure 5:**
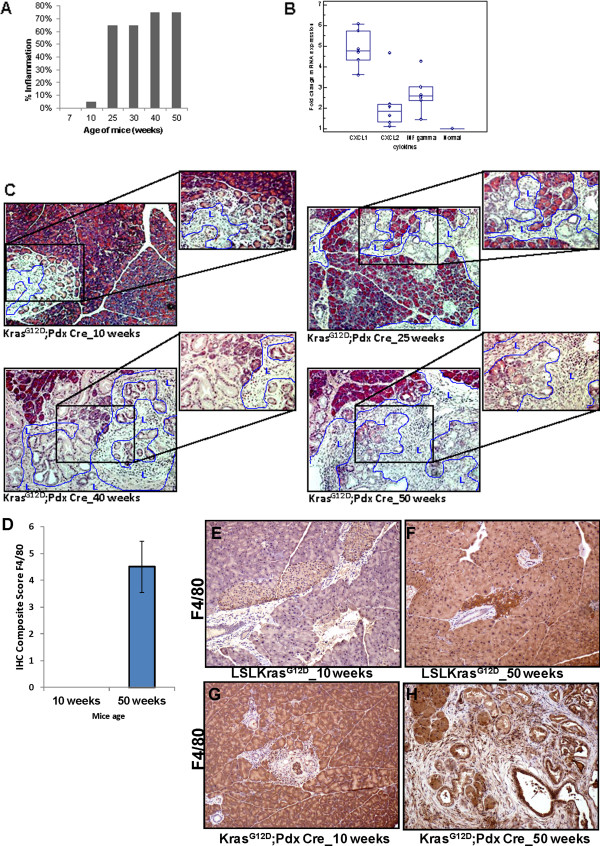
**Inflammation during the progression of pancreatic cancer in Kras**^**G12D**^**mouse model.** (**A**) Percentage of inflammation in the pancreas of 7 to 50 weeks old Kras^G12D^;Pdx1-Cre mice evaluated on H&E stained tissue sections. (**B**) Expression of mRNA of the inflammatory cytokines/chemokines *IFNγ*, *CXCL1* and *CXCL2* in the pancreas of 50 weeks old Kras^G12D^;Pdx1-Cre mice compared to LSLKras^G12D^ (i.e. normal, unfloxed) animals. (**C**) Infiltration of lymphocytes into the pancreas of Kras^G12D^;Pdx1-Cre mice. Lymphocytes (L) that infiltrated into the pancreas of Kras^G12D^;Pdx1-Cre mice are shown within the blue boundaries in the H&E stained tissues. Light microscopic pictures are magnified 200x for each age group. **D**) Composite scores of pancreatic tissues of Kras^G12D^;Pdx1-Cre mice stained with F4/80 antibody for macrophages. (**E**, **F**, **G**, **H**) F4/80 marker expression for macrophages during the progression of pancreatic cancer in Kras^G12D^;Pdx1-Cre mouse model was analyzed by IHC.

Oncogenic *Kras* has been implicated in the activation of the NF-κB pathway which induces inflammatory responses in PC [30] and the production of cytokines from tumor cells which result in the generation of a pro-inflammatory tumor microenvironment in the bronchiolar epithelium [31]. As mucin genes are known to be regulated under inflammatory conditions [32-34], we wanted to investigate whether immune infiltration occurred early during PC development. There was no inflammation in the pancreas at 7 weeks of age (postnatal), but at 10 weeks of age, mild inflammation reaction was observed in 5% of the pancreatic tissues (Figure 5A). Subsequently, chronic inflammation was observed in 65% of the pancreatic tissues in 25-30 weeks old Kras^G12D^;Pdx1-Cre mice which increases to 75% by 40-50 weeks of age with a strong desmoplastic reaction (Figure 5A). This inflammation scoring was further corroborated with the infiltration of macrophages (F4/80) in the cancer tissue (Figure 5G and 5H) with a composite score of 4.5 (p < 0.05) (Figure 5D) compared to 10 weeks of age (Figure 5E and 5F), where mostly PanIN I were observed.

Expression of inflammatory cytokines/chemokines such as *IFN-γ*, *CXCL1* and *CXCL2* were measured by performing real time-PCR using total RNA isolated from mouse pancreas collected at 50 weeks of age. We observed a significantly higher expression of *CXCL1* (p < 0.00013), *CXCL2* (p < 0.085) and *IFN-γ* (p < 0.0062) in Kras^G12D^;Pdx1-Cre animals compared to LSLKras^G12D^ control animals (Figure 5B). Correspondingly, an increased infiltration of lymphocytes in pancreatic tissues of Kras^G12D^;Pdx1-Cre mice correlated with the increased inflammation and increased inflammatory cytokines detected in the pancreas of Kras^G12D^;Pdx1-Cre mice (Figure 5C).

## Discussion

PC is an extremely lethal disease, with a five year survival rate of less than 5% and a median survival period of 5-6 months. At the time of diagnosis, PC metastasizes to regional lymph nodes and distant organs and responds poorly to current chemo- and radiation therapies resulting in a high recurrence rate [1-3]. The poor prognosis and weak therapeutic responses are a consequence of late diagnosis of the majority of PC patients, primarily due to lack of early symptoms and reliable early diagnostic markers [2]. Therefore, there is an urgent need to identify specific early biomarkers for early diagnosis and molecular targets for effective treatment of PC.

Previous studies done in human tissues have indicated an aberrant overexpression of various mucins in several epithelial malignancies including pancreatic, ovarian and lung cancers [7,10]. Thus, not surprisingly, their potential in the diagnosis and targeted treatment of PC has been suggested and tested over the last decades [35,36]. In cancer cells, mucins play an important role in cell growth, differentiation, transformation, adhesion, invasion and immune evasion [5,8,20]. In human PC tissues, MUC1, MUC4, and MUC5AC are aberrantly upregulated and their expression has been linked to the progression and poor prognosis of the disease. However, due to the late diagnosis of PC, the status of mucin expression in the earliest stages of the disease remains unknown.

Genetically engineered mouse models can facilitate the discovery of tumor biomarkers in order to design powerful techniques to diagnose, treat, and monitor therapeutic efficacy in cancer patients more effectively [37]. Mouse Muc1 shares 34% homology with human MUC1 in the tandem repeat region mainly sharing threonine, serine and O-linked sugars but it is 87% homologous at transmembrane and cytoplasmic regions. Due to high degree of conservation in the promoter region (74%), the patterns of expression of mouse Muc1 is quite similar to human MUC1 [25]. Similarly, the mouse and human MUC4 have identical exon/intron structure [38]. Further, human MUC4 homology analysis with mouse, dog, rat, and chicken Muc4 revealed that NIDOgen-like (NIDO), Adhesion associated domain of MUC4 and Other Proteins (AMOP), von Willebrand factor D (vWD), Epidermal Growth Factor (EGF), transmembrane (TM), and cytoplasmic tail (CT) domains are highly conserved across the species suggesting that individual domains evolved from common ancestral domains and share common functions [22]. In the case of mouse Muc5AC (located on chromosome 7), it shares 52% homology with human MUC5AC (located on human chromosome 11) and TATA box regions in both the species are fully conserved [39]. Because mucin genes are conserved between humans and mice, such mouse models provide a unique opportunity to examine the expression profile and possibly functional role of mucin genes at the earliest stages of the disease.

We used a well characterized Kras^G12D^;Pdx1-Cre spontaneous PDAC mouse model, which recapitulates human PC genetically, histologically and pathologically [40], to investigate if the expression pattern of murine mucins (i.e. Muc1, Muc4 and Muc5AC) mirrors the altered mucin profile of the human disease. The Kras^G12D^;Pdx1-Cre genetically engineered mouse PDAC model was chosen over other spontaneous PDAC models because it recapitulates the full spectrum of human PanIN lesions, which are recognized as early events in PC. Moreover, mass spectrometry proteomics analysis in this mouse model identified a distinct serum proteome having preinvasive PanIN lesions compared to healthy controls [40], emphasizing its utility as a suitable platform to understand early stages of PC that may lead to the optimization of diagnostic and therapeutic techniques against this malignancy.

MUC1 is a transmembrane mucin with basal level expression in normal epithelial cells lining various organs including the pancreas. It has been shown to be overexpressed and aberrantly glycosylated in PC and play a role in the invasion and metastasis of PC [6,8,19]. Overexpression of MUC1 has been observed during the early stages of PC development, with a subsequent increase in expression in invasive carcinoma, both in humans and p48; Kras^G12D^; MUC1.Tg mouse model [18,41]. Similarly, IPMNs like lesions from Kras^G12D^;TGFÎ±;Pdx-1-Cre transgenic mice showed elevated Muc1 and Muc5AC expression at 3 months of age [42] and recent reports also revealed that Kras^G12D^;P48-Cre; Muc1KO mice had slower tumor progression and metastasis compared to both Kras^G12D^;P48-Cre and Kras^G12D^;P48-Cre; MUC1 transgenic animals [43]. On the other hand, Muc1 null mice are phenotypically normal and exhibit normal reproduction and survival rate [17]. Previous studies in human pancreatic tissues also reported an increase in MUC1 expression which correlated with grade of PanIN lesions and PDAC [44]. In our study, mRNA and protein levels of Muc1 progressively increased from 10 weeks to 50 weeks of age in the pancreas of Kras^G12D^;Pdx1-Cre mice compared to unfloxed LSLKras^G12D^ mice, and correlated with the development of PDAC from PanIN precursor lesions (Figure 2). Thus, the expression of Muc1 in the Kras^G12D^;Pdx-1-Cre spontaneous PDAC progression model corroborates its resemblance with the human disease.

MUC4 is a high molecular weight, type I transmembrane glycoprotein that is overexpressed in PC but absent in normal pancreas and chronic pancreatitis [10]. Although previous studies in human specimens have shown an increased expression of MUC4 in PC progression and metastasis [10,11], it remains unknown if MUC4 overexpression is an early event in PC. MUC4 expression has been observed in precursor PanIN lesions in clinical samples [45], which is suggestive of, but not a definitive proof of MUC4 overexpression as an early event in PC. In the present study, we observed that Muc4 mRNA and protein levels increased progressively from 10 weeks of age, which is when we observed the appearance of PanIN I lesions and continued to increase up to 40 weeks of age where we observed advanced PanIN III lesions (Figure 3). Our findings establish that Muc4 expression is indeed an early event in PC progression, which recapitulates the MUC4 expression profile in human PC. Future studies using Muc4 knock out and MUC4 transgenic animals on the Kras^G12D^ murine background will help delineate the molecular mechanisms and contribution of Muc4 in PC progression and metastasis. Nonetheless, the present study establishes the suitability of Kras^G12D^ model for evaluating the potential of Muc4 as an early diagnostic marker and therapeutic target.

The expression of the gel-forming secretory mucin MUC5AC in human PC increases progressively with the increase in grade of PanIN lesions and PDAC, whereas it is undetected in normal pancreas [19,29]. Similar to the expression of the transmembrane mucins MUC1 and MUC4, MUC5AC expression has also been related to PC progression [46] and it is associated with a shorter survival period of PC patients [13]. In the present study, Muc5AC expression in the pancreas of Kras^G12D^;Pdx1-Cre spontaneous PDAC mice increased progressively from 10 to 50 weeks of age (Figure 4) as compared to unfloxed LSLKras^G12D^ mice, corroborating studies of the human disease. It is important to emphasize the particular usefulness of the detection of Muc5AC in early lesions of PC, as its secretory nature is advantageous for non-invasive serum based diagnostics.

Previous studies with human tissues have implicated *Kras* activation in rigorous inflammatory responses in PC, mainly by activating the NF-κB pathway [30]. In agreement with these studies, recent studies reported the observation of proinflammatory responses in the Kras^G12D^;PdxCre spontaneous PDAC mouse model, which suggested that chronic inflammation is indeed a precursor and potentially a key factor in promoting PC [47]. These studies suggested that constitutive NF-κB activation and inflammatory responses induced by oncogenic *Kras* are one of the earliest events in PC development. Mucins are known to be transcriptionally regulated by inflammatory cytokines like IFN-γ (MUC4) [32] and neutrophil elastase (MUC1 and MUC5AC), which is a serine proteinase secreted by neutrophils during inflammation [33,48]. Moreover, a recent study demonstrated that glycosylation of mucins can be altered in response to proinflammatory conditions in PC cells [34]. Given the functional and pathological significance of MUC1, MUC4 and MUC5AC in PC progression and their regulation by inflammatory environment in the human disease, we analyzed the inflammation in the pancreas of Kras^G12D^;Pdx1-Cre mice. Increased inflammation in the pancreas of Kras^G12D^;Pdx1-Cre spontaneous PDAC mice correlated with an increase in inflammatory cytokines/chemokines such as *INFγ* (p < 0.0062), *CXCL1* (p < 0.00014), *CXCL2* (p < 0.08) and lymphocyte (Figure 5C) and macrophage infiltration (Figure 5D, G-H). These results correlate with an increase in the expression of Muc1, Muc4 and Muc5AC in the pancreas of Kras^G12D^;Pdx1-Cre spontaneous PDAC mouse model, suggesting a possible link between inflammation and mucin expression, which further recapitulates the studies done in the human disease.

## Conclusions

Our studies are the first to establish that Kras^G12D^;Pdx1-Cre mouse model recapitulates the alterations in mucin expression observed during the progression of human PC. Although *Kras* was the first oncogene identified to play a critical role in PDAC development, its activity is involved in PanIN initiation but not sufficient to induce PDAC by itself [49]. Inflammatory signaling pathways triggered by oncogenic *Kras* may synergize with other critical molecules to upregulate mucin expression during the early development of PC. The present study provides the basis to investigate the functional role of specific mucins in PC initiation and progression by generating corresponding transgenic and knockout animals and crossing them with spontaneous models of PC. It will also be interesting to study the fate of mucin expression in the mouse pancreas in response to inflammatory stimuli like smoking and alcohol that induce pancreatic pathologies. Our studies also establish that Kras^G12D^;Pdx1-Cre mouse model is ideally suited to investigate mucin-based biomarkers and targeted therapies for PC.

## Competing interests

The authors have no conflict of interest with any company or financial organization.

## Authors' contributions

Drs SKB, SR and MJ designed experiments, critically evaluated the work and took overall supervision in the preparation of manuscript, SR performed animal crosses generated progression model and collected tissues with help of MB, SKaur, MPP and PS. MPT, DH performed immunohistochemistry. SKumar, DH and PD isolated RNA and performed real time PCR, SR, MPT and MAM analyzed data and prepared manuscript, SLJ served as the certified pathologist for scoring of H&E as well as scoring of IHC slides. KW provided guidance for the animal work. All authors read and approved the final manuscript.
